# Spatiotemporal intermittence effect on periphyton microbial communities in a tropical drying river network

**DOI:** 10.1093/ismeco/ycag084

**Published:** 2026-04-16

**Authors:** Milena D Campaña, Daniela Rosero-López, María de Lourdes Torres, Daniel Escobar-Camacho, J L Weissman, Juan José Guadalupe, Darío X Ramírez-Villacís, Jordan Karubian, Andrea C Encalada

**Affiliations:** Laboratorio de Ecología Acuática, Universidad San Francisco de Quito, Diego de Robles S/N, Quito, Pichincha Province, 170902, Ecuador; Laboratorio de Ecología Acuática, Universidad San Francisco de Quito, Diego de Robles S/N, Quito, Pichincha Province, 170902, Ecuador; Global Research and Solutions Center, Universidad San Francisco de Quito, Diego de Robles S/N, Quito, Pichincha Province, 170902, Ecuador; Global Research and Solutions Center, Universidad San Francisco de Quito, Diego de Robles S/N, Quito, Pichincha Province, 170902, Ecuador; Laboratorio de Biotecnología Vegetal, Universidad San Francisco de Quito, Diego de Robles S/N, Quito, Pichincha Province, 170902, Ecuador; Laboratorio de Ecología Acuática, Universidad San Francisco de Quito, Diego de Robles S/N, Quito, Pichincha Province, 170902, Ecuador; Global Research and Solutions Center, Universidad San Francisco de Quito, Diego de Robles S/N, Quito, Pichincha Province, 170902, Ecuador; Department of Ecology and Evolution, Stony Brook University, Stony Brook, NY 11794, New York, United States; Institute for Advanced Computational Science, Stony Brook University, Stony Brook, NY 11794, New York, United States; Laboratorio de Biotecnología Vegetal, Universidad San Francisco de Quito, Diego de Robles S/N, Quito, Pichincha Province, 170902, Ecuador; Laboratorio de Biotecnología Vegetal, Universidad San Francisco de Quito, Diego de Robles S/N, Quito, Pichincha Province, 170902, Ecuador; Department of Ecology and Evolutionary Biology, Tulane University, New Orleans LA 70118, United States; Fundación para la Conservación de Los Andes Tropicales, Quinindé city, Esmeraldas Province, 080451, Ecuador; Laboratorio de Ecología Acuática, Universidad San Francisco de Quito, Diego de Robles S/N, Quito, Pichincha Province, 170902, Ecuador; Global Research and Solutions Center, Universidad San Francisco de Quito, Diego de Robles S/N, Quito, Pichincha Province, 170902, Ecuador

**Keywords:** microbial communities, amplicon sequence variants (ASV), tropical drying river networks, diversity

## Abstract

Drying river networks (DRNs) are globally expanding ecosystems where hydrological intermittence, including surface-flow cessation and habitat fragmentation, profoundly shapes biodiversity, ecological functions, and biogeochemical processes. Despite growing knowledge, the role of microbial communities inhabiting periphyton in tropical DRNs remains poorly understood. Here, we conducted the first spatiotemporal metabarcoding assessment (16S & 18S V4 amplicon sequencing) of bacteria, phototrophs, and fungi in stream periphyton across the Cube Drying River Network, located in Ecuador’s Chocó–Darién biodiversity hotspot. We sampled 20 reaches spanning perennial and intermittent sites during six campaigns across an intermittence gradient. Microbial α-diversity increased with drying, peaking at the highest intermittence (45%), with phototrophs and fungi exhibiting significant gains compared to wetter phases. Community β-diversity declined as intermittence intensified, indicating homogenization of assemblages under sustained hydric stress and restricted dispersal due to disconnection of surface flow. Variation partitioning showed that major dissolved constituents (e.g., Ca^2+^, Mg^2+^, NO₃^−^, TOC) explained ~6% of compositional shifts, while environmental variables and trace elements accounted for smaller fractions. Co-occurrence network analyses revealed that interactions among microbial groups intensified with drying, culminating in highly connected yet low-modularity networks, which suggests reduced resilience under prolonged intermittence. Our results contrast with patterns reported in temperate and arid systems, where drying typically reduces microbial diversity, underscoring the particular dynamics of tropical DRNs, which are dominated by isolated pools and incomplete drying. These findings underscore the role of periphyton as critical microbial hubs that regulate ecosystem functioning under fluctuating flow regimes and emphasize the urgent need to integrate microbial dynamics into global assessments of river network resilience in the face of climate change.

## Introduction

Drying river networks (DRNs) are freshwater systems composed of river and stream reaches that periodically dry or cease to flow and are among the world’s most widespread and dynamic freshwater ecosystems [1]. They experience recurrent hydrological transitions between wetting, low-flow fragmentation and disconnection, drying, and re-wetting phases, driven by both natural and anthropogenic factors [2, 3]. Under current climate change scenarios, the frequency and extent of drying are projected to increase [4, 5]. Local α-diversity generally declines in response to drying due to the loss of species that are unable to tolerate or recover from desiccation [5, 6]. In contrast, as drying creates a mosaic of habitats, β-diversity increases [6]. However, responses can be region-specific and modulated by the severity and spatiotemporal pattern of intermittence [5, 6]. In DRNs from arid, semi-arid, and temperate biomes, periphyton microbial communities experience hydric stress and subsequently senesce, enter dormancy, or die in response to drying [7–9]. This wealth of responses between wet and dry states [10], ranging from cells to biofilms, underscores the importance of deciphering α and β-diversity to understand the stability of periphyton communities and, consequently, ecosystem functions [7, 11]. Microbial communities in stream periphyton (biofilm) create the most important interface between substrate and flow, acting as a “microbial skin” that, unlike a static or resilient barrier, mediates solute exchange and biological interactions [12–14]. Despite continuously resetting hydrological conditions, periphyton contributes significantly to the productivity of all streams [7, 11, 13, 14]. Microbial communities in the periphyton drive organic matter decomposition [15, 16], nutrient cycling [17], and whole-stream respiration [18], and they rely on light availability and oxygen, which vary with water movement and level [19–21].

In this context, we performed an in-depth spatiotemporal analysis using a metabarcoding approach (16S rRNA gene and 18S rRNA V4 region amplicon sequencing) to assess the major taxa present in stream periphyton, including bacteria, phototrophs, and fungi in a global biodiversity hotspot. The Chocó–Darién ecoregion is characterized by highly dynamic hydroclimatic conditions, with natural intermittence occurring under pronounced seasonality [22]. This global biodiversity hotspot presents high endemism while increasing threats [23]. Although various biological groups have been studied here, the diversity of microbial communities has received comparatively little attention, and no research has specifically addressed the effects of fragmentation-driven intermittence on streams’ periphyton of tropical DRNs. We therefore aimed to determine how spatiotemporal intermittence modulates periphyton microbial diversity, environmental filtering, and co-occurrence in a tropical DRN. Based on existing research of periphyton in intermittent rivers and ephemeral streams experiencing complete drying [-, 11, 20, 24, 28], we proposed the following hypotheses: **H1**: The α-diversity of microbial communities in the periphyton will decrease due to hydric stress, and the β-diversity will increase in drying conditions due to a mix of available microhabitats. **H2:** Hydrological variables and water chemistry will act as secondary modulators of periphyton microbial community composition under variable intermittence conditions, exerting a weaker influence than intermittence itself; and **H3:** The interactions between bacteria, phototrophs, and fungi communities in the periphyton will decrease as drying conditions increase.

This study helps fill knowledge gaps on the effects of drying in tropical DRNs, highlighting the role these networks play in shaping regional patterns of intermittence and contributing to a broader global understanding of these processes [4, 10, 29]. By analyzing the spatiotemporal responses of basal organisms (phototrophs, bacteria, and fungi) to drying, we aim to provide insights into the role that local and regional diversity could play in biogeochemical processes such as nutrient and trace elements cycling, ecological functions like organic matter decomposition, and overall ecosystem services such as carbon sequestration in tropical DRNs.

## Materials and methods

### Study area, sampling design, and intermittence at the network scale

**Figure 1:**
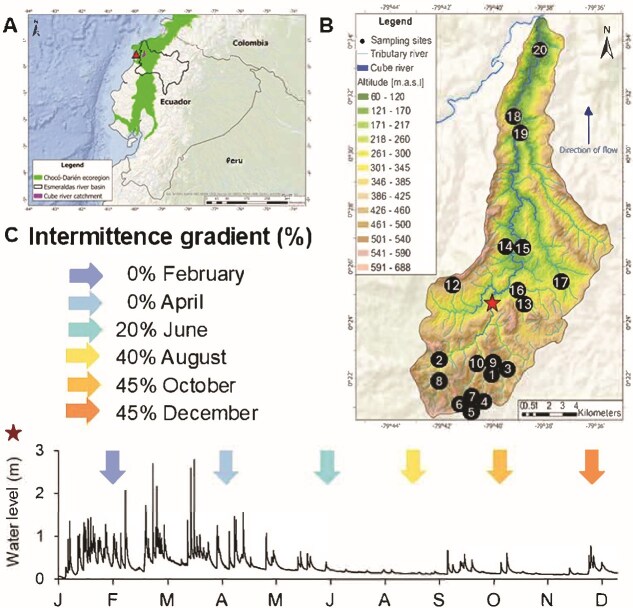
A. Study area (triangle) in the Esmeraldas River basin along the Chocó-Darién ecoregion; B. The Cube Drying River network showing sampling sites numbers (circles) (n = 20) distributed along the elevational gradient; C. The intermittent gradient (%) calculated for each sampling campaign (month), during the 2021 hydrological period (star, measured at site 11), corresponding to the proportion of dry sites and sites with disconnected pools to the total number of sites (calculated from Eq 1), the scheme color represents the progress of drying from the wet to the dry season.

The study area is the Cube Drying River Network (DRN), a catchment of 165.15 km^2^ located in the Chocó-Darién ecoregion of Ecuador, at coordinates −79.66441 W, 0.37817 S; −79.63394 W, 0.56257 S (Fig. 1A). The Cube River flows from south to north from 532 to 57 m of altitude (Fig. 1B), and drains into the Viche River, a tributary of the Esmeraldas River Basin in the Pacific Lowlands [30]. The Cube DRN experiences a highly seasonal climate, characterized by a pronounced wet season from January to May and a dry season from July to December (Fig. 1C, Fig. S1) [31]. For sampling, we selected 20 stream reaches, referred to as “sites,” distributed across the headwaters, the main stem, and near the mouth, and capturing diverse land uses along the altitudinal gradient. Using local information, we selected sites known for drying for part of the year (n = 9), which matched the headwaters and a first-order tributary. Perennial flow sites are located in the middle and lower parts of the network (n = 11) (Fig. 1B). We conducted six sampling campaigns in February, April, June, August, October, and December of 2021 (Fig. 1C) aimed to capture the intermittence gradient throughout the seasons [32]. We assessed hydrogeographically the degree of drying for each sampling campaign as the Intermittence gradient (%) at the network scale. To this end, we calculated the proportions of dried sites and sites with disconnected pools observed during sampling. We divided them by the total number of sites (n = 20) to obtain a hydrogeographical Intermittence gradient (%) (Equation 1).


$$ Intermittence\ gradient\ \left(\%\right)=\frac{\# Dry\ sites+\# Disconnected\ Pools\ sites}{Total\# of\ Sites} $$

This metric captures flow fragmentation and surface water disconnection as key expressions of intermittence that do not require complete desiccation. Each sampling campaign was identified by the Intermittence gradient (%) and not simply the month (i.e., February and April; October and December), therefore limiting temporal confounding and allowing us to distinguish hydrological states even when occurring during the same season (Fig. 1C). We employed a degraded color scheme to represent the sampling campaigns (month), and we maintained this pattern throughout the entire manuscript.

In each sampling campaign, we measured stream/river hydrological variables (Table 1, Table S1), including discharge, stream width, mean depth, bank-full width, among others, in five equidistant transects that covered the entire stream reach, named as “site.” We measured In Situ the pH, temperature, conductivity, and dissolved oxygen using a multiparameter sonde (YSI, ProDSS®). At the upstream part of the stream reach, we collected two replicates of 1 L water samples and preserved them with 2 mL of 0.1% HNO_3_ for further analysis at the Core-Lab at the Universidad San Francisco de Quito. We analyzed 12 hydrological variables, nine major (Table 1, Table S2), and eight minor chemical constituents (Table 1, Table S3).

**Table 1:** Example results from environmental variables and major and minor constituents present in water analyzed in the 20 sampling sites in the Cube Drying River Network. Mean ± SD corresponds to six sampling campaigns carried out in 2021. Tables S1, S2, and  S3 provide a complete list of variables and results.

	***Hydrological variables***						***Major constituents***											***Minor constituents***				
**Site**	**Altitude**	**Discharge (l/s)**			**Temp. (°C)**		**pH**			**DO (ppm)**			**Cond. (uS/cm)**			**TOC (ppm)**		**Pb (ppm)**			**V (ppb)**	
	**(m.a.s.l.)**	**Mean**	**±SD**		**Mean**	**±SD**	**Mean**	**±SD**		**Mean**	**±SD**		**Mean**	**±SD**		**Mean**	**±SD**	**Mean**	**±SD**		**Mean**	**±SD**
1	493	4.19	4.7		21.55	0.62	7.3	0.4		7.55	0.79		112.58	44.90		3.13	1.82	4.83	1.29		1.19	0.16
2	334	45.01	48.9		28.72	0.62	7.7	0.3		8.79	0.17		136.81	48.37		2.71	1.21	5.72	2.52		1.91	0.72
3	475	1.71	2.3		21.01	1.86	7.0	0.5		5.24	1.84		60.11	20.06		2.13	1.32	5.50	1.75		1.42	0.46
4	533	18.59	27.4		22.11	0.86	7.3	0.6		7.40	0.81		101.92	46.24		2.25	1.00	5.68	1.75		1.72	0.71
5	516	8.97	9.1		21.64	0.75	7.6	0.2		8.43	0.15		105.94	35.21		2.08	0.54	5.51	2.00		1.28	0.17
6	479	6.29	9.3		21.92	0.84	7.6	0.4		8.74	0.47		103.52	29.99		1.61	0.53	5.04	2.26		1.46	0.34
7	457	24.06	33.9		28.28	0.65	7.5	0.3		7.78	0.62		181.86	167.98		1.73	0.17	4.92	2.18		1.81	1.08
8	321	37.41	54.2		29.30	0.59	7.9	0.2		8.96	0.20		226.18	112.07		1.80	0.61	6.67	2.37		1.85	0.60
9	320	113.92	111.9		22.82	0.74	7.8	0.5		9.02	0.46		174.97	72.63		2.10	1.00	5.18	1.95		2.11	0.94
10	266	562.48	1094.7		23.60	0.62	7.9	0.5		9.65	0.78		199.47	77.72		2.14	1.28	5.64	2.71		2.18	0.86
11	185	286.22	300.4		24.24	0.86	7.7	0.7		8.97	0.46		225.98	72.26		2.24	1.07	5.49	2.88		2.34	1.02
12	372	28.97	53.7		23.03	0.74	7.5	0.3		6.70	0.79		262.08	28.76		1.32	0.84	19.73	20.35		4.63	2.15
13	208	295.43	526.1		24.34	1.25	7.6	0.3		8.06	0.29		133.44	36.59		2.43	0.73	5.68	2.53		2.07	1.31
14	135	7.76	6.4		25.83	0.64	7.7	0.0		8.50	0.95		457.63	99.76		1.65	0.61	5.16	2.80		1.22	0.12
15	126	1151.46	1566.3		25.58	0.85	7.9	0.4		9.24	0.69		338.96	78.78		2.01	0.37	6.82	3.34		1.81	0.82
16	201	260.19	475.3		24.52	0.80	8.0	0.6		9.23	1.03		336.46	135.46		2.14	1.07	6.84	3.50		2.34	1.14
17	196	206.52	396.5		24.09	0.48	7.7	0.3		8.47	0.67		434.24	136.20		1.81	0.82	5.49	4.30		2.69	2.95
18	82	356.92	590.9		25.34	0.84	8.2	0.5		9.81	1.42		461.79	146.19		1.93	0.72	5.76	3.36		2.28	1.30
19	77	2896.23	5465.3		26.58	0.66	8.1	0.3		10.49	2.17		450.93	102.42		1.80	0.64	7.27	5.48		2.30	0.95
20	57	2838.85	3607.3		26.06	1.97	8.1	0.3		9.58	1.47		432.57	168.21		1.79	0.46	6.66	4.43		2.75	2.03

### Periphyton collection and DNA extraction

We collected periphyton in streams by randomly selecting six cobbles per site, trying to match a proportion of three from rapids and three from pools. When rapids were unavailable due to drying and only disconnected pools remained, all rocks were collected from the pools. We collected them using nitrile gloves by keeping the biofilm facing upwards, in a pre-cleansed plastic tray. In the field, we followed the standard protocol by scraping periphyton from the exposed surface using a plastic bristle brush, which had been previously rinsed with 1% HCl [33]. We used 160 ml of distilled water to rinse the slurry in the plastic tray. We collected two replicates of 40 mL samples in sterile Falcon tubes using nitrile gloves. All instruments were sterilized under a Bunsen flame and rinsed with 1% HCl and distilled water to avoid site cross-contamination. The samples were labeled and kept at −20°C until analysis. Once in the Plant Biotechnology Laboratory at Universidad San Francisco de Quito, we thawed the samples and then centrifuged 7.5 mL of each sample at 13000 RCF for 20 minutes. Then, 500 μL of the pellet was recovered for DNA extraction [34].

All periphyton samples were initially processed using the DNeasy PowerSoil Pro DNA Isolation Kit to maintain methodological consistency. For a subset of samples that yielded low DNA concentration or poor purity, likely due to the high extracellular polymeric substance content and mineral matrix characteristic of stream biofilms, a phenol–chloroform extraction was applied to ensure sufficient DNA for amplification and sequencing.

#### Sequencing and bioinformatics

Sequencing of amplicons from 64 samples was performed using an Illumina MiSeq PE300 platform (Illumina, USA). The extracted genomic DNA was used as a template in PCR amplification of bacterial 16S (V3-V4) rRNA and eukaryotic 18S (V4) rRNA genes. Primers ID (5′-3′) used in this study for 16S (V3-V4) were Bakt_341F: CCTACGGGNGGCWGCA and Bakt_805R: GACTACHVGGGTATCTAATCC [35], and for 18S (V4) were V4F: CCAGCAGCCGCGGTAATTCC and V4R: ACTTTCGTTCTTGATTAA [36]. Taxonomy was assigned using SILVA 138.1 for 16S rRNA genes (August 27, 2020) and PR2 5.0.0 for 18S rRNA genes (April 6, 2023).

### Statistical analysis

#### Data filtration and community composition

For further analysis, we performed rarefaction using the “rarefy_even_depth” function from the phyloseq package (31 783 read depth) (Fig. S2). Then, we performed further filtration to keep only high-abundance amplicon sequence variants (ASVs) (> 0.1% relative abundance) (Table S4). With ASVs data, we calculated α-diversity using the Chao1 Index and an unpaired t-test to assess significant differences. To visualize, only orders with at least 5% relative abundance are identified. The abundance of ASVs was plotted in a heatmap using logarithmically transformed ASV abundance data with the pheatmap package version 1.0.12 in R [37], where hierarchical clustering was performed using Pearson correlations and the Ward method.d2 algorithm. To confirm differences in community composition along the intermittence gradient, we first quantified β-diversity using Bray-Curtis dissimilarities. These dissimilarities were then used in a constrained analysis of principal coordinates (CAP), where the ordination was constrained by the intermittence gradient (%) and conditioned by the sites’ positions. We tested whether the ecological patterns observed along the intermittence gradient were statistically significant using a Permutational Multivariate Analysis of Variance (PERMANOVA) with the R package “vegan.”

#### Multivariate analysis

To assess the effect of hydrological variables (Table 1, Table S1) as well as water chemistry separated into major (e.g., Fe^2+^, PO_4_^3−^, NO_3_^−^) (Table S2) and minor constituents (e.g., Pb, Mo, Ni) (Table S3), on the microbial community composition (β-diversity) across the intermittence gradient, we ran Mantel tests [38], using Euclidean distances for the hydrological variables and minor and major constituents dissolved in water. We used the scale function from base R and standardized the data into Z scores [39]. We performed a variation partitioning analysis by standardizing the data using the Hellinger method [40] with the “decostand” function from the vegan package in R to understand further how these variables influenced ASV abundance.

**Figure 2:**
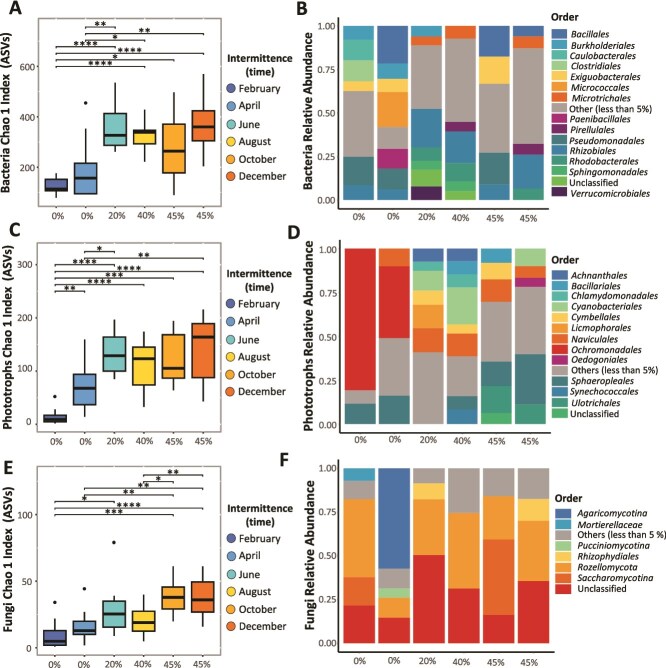
Spatiotemporal microbial community response to the intermittence gradient (%) in the cube Drying River network: Α-diversity (Chao1 index) and the relative abundance (%) of most abundant orders (>5%) of ASVs (amplicon sequence variance) for (A, B) bacteria, (C, D) phototrophs, and (E, F) and fungi. Statistical significance is represented by the following levels: ^*^0.05, ^**^0.005, ^***^0.0005, and ^****^ < 0.00001 (table S5).

#### Ecological co-occurrence network analysis

To assess microbial community interactions, we inferred co-occurrence networks from bacterial and eukaryotic data (16S rRNA gene and 18S rRNA V4 region, including bacterial, phototrophic, and fungal ASVs) using dissimilarity matrices and SparCC. Which estimates correlations from compositional data, retaining only the 1% strongest positive correlations to emphasize robust associations and ensure comparability across the intermittence gradient (%).

A unique network was constructed for each intermittence condition (month) using the Meinshausen and Bühlmann (mb) method [41] with previously filtered data. The following ASVs were filtered out for each network: ASVs with null abundance, ASVs with an average of two reads, and ASVs not present in at least half of the samples. Edges were further filtered to keep only the following interactions: bacteria-phototrophs, phototrophs-fungi, and fungi-bacteria. Additionally, only 1% of the top interaction strength was plotted. We implemented a fast-greedy clustering algorithm to detect clusters within each network [42, 43]. We calculated topological measures, including edge and node counts, diameter, cluster number, and modularity. Modularity indicates the level of interconnection and the potential of networks being vulnerable or resilient to disturbances. All networks were plotted using igraph R package [44].

## Results

The Cube DRN yielded a spatiotemporal distribution of streams with dry sites and sites with disconnected pools. During the wet season (February and April), the Cube DRN had no dry sites or disconnected pools, resulting in 0% intermittence for both sampling campaigns. This differentiation was evident in the intensity of the flash floods represented in the hydrograph from site 11 (Fig. 1C). As flash floods decreased after April, dry sites and sites with disconnected pools started to appear in the network, reaching 20% intermittence in June and 40% intermittence in August. With the progression of the dry season, 45% of the Cube DRN dried and/or disconnected in October and December. At the river network scale, after rarefaction, we detected a total of 43 768 ASVs for bacteria (16S rRNA gene), 4701 for phototrophs, and 1726 for fungi (18S rRNA V4 region) in stream periphyton.

### Periphyton microbial communities’ composition in the intermittence gradient

#### Effect on α-diversity and relative abundance

Overall, periphyton microbial communities in the Cube DRN responded positively to the increase in intermittence (R^2^ = 0.686) (Fig. 2). The α-diversity of bacterial ASVs, ~300, exceeded that of phototrophs, ~150, and fungi, ~25, with phototrophs exhibiting greater ASV diversity than fungi. The α-diversity of bacteria was significantly higher at 45% intermittence (December) than diversity at 0% intermittence in February (t = −9.53, df = 23, *p < 0.00001*) and April (t = −3.48, df = 13, *p = 0.0042*) but not statistically different than diversity at 20% (June), 40% (August) and 45% (October) intermittence (Table S5) (Fig. 2A). The relative abundance of bacterial orders changed with the intermittence gradient. A more complex community composition, comprised of seven orders with relative abundance >5%, occurred at 20% intermittence (June) compared to the composition at 45% intermittence (October), with only four orders (Fig. 2B). The group of “Other,” with relative abundance <5%, increased from 40% intermittence onward. Specific orders like *Clostridiales* with relative abundance of 12.15% and *Caulobacterales* with 11.88% were present at 0% intermittence (February), whereas *Micrococcales* with 20.08% relative abundance and *Paenibacillales* with 11.35% were present at 0% intermittence (April). The order of *Verrucomicrobiales* with 7.81% only appeared at 20% intermittence (Fig. 2B). The α-diversity of phototrophs also increased with drying and reached the highest Chao1 diversity at 45% intermittence (December) (Fig. 2C). The α-diversity of phototrophs at 0% intermittence (February) was significantly lower than all diversities across the intermittence gradient. At 0% the low α-diversity of phototrophs was highly significant to diversities at 20% (June) (t = −8.85, df = 12, *p < 0.00001*) and at 45% (December) intermittence (t = −8.4, df = 21, *p < 0.00001*) (Table S5). Differences in α-diversity between 0% (April) and 45% (October) intermittence were not statistically significant (Fig. 2, Table S5). In the phototrophs, the community compositions at 0% intermittence (February and April) were constrained to three and four orders, respectively. The order *Ochromonadales* with relative abundance of 80.86% and 40.99% was dominant only at 0% intermittence in February and April, respectively (Fig. 2D). A more complex community composition was observed at 40% intermittence than at the extremes of the intermittence gradient. The order *Synechococcales* persisted across the intermittence gradient, except at 0%. Contrastingly, the order *Cyanobacteriales* appeared at 20%, 40%, and at 45% intermittence with a peak of relative abundance in August (21.04%), while the order *Oedogoniales* with a relative abundance of 5.11% was observed at 45% intermittence (December) but present within the Others group across the gradient (Fig. 2D). Fungi α-diversity was significantly higher at 45% intermittence (December) than at 0% intermittence (February) (t = −5.58, df = 20, *p < 0.00001*) (Table S5) (Fig. 2E). The diversity of fungi at 40% intermittence (August) was significantly lower than at 45% intermittence in October (t = −2.97, df = 13, *p = 0.0112*) and December (t = −3.73, df = 22, *p = 0.0011* (Table S5). For the community composition of fungi, the relative abundance of orders was similar across the intermittence gradient, except at 0% intermittence (April), where *Agaricomycotina* was the dominant order. The order *Pucciniomycotina* was only present with relative abundance above 5% at 0% intermittence (April), but also with 2% relative abundance in 0% intermittence (February) (Fig. 2F).

#### Amplicon sequence variance clustering and β-diversity

**Figure 3:**
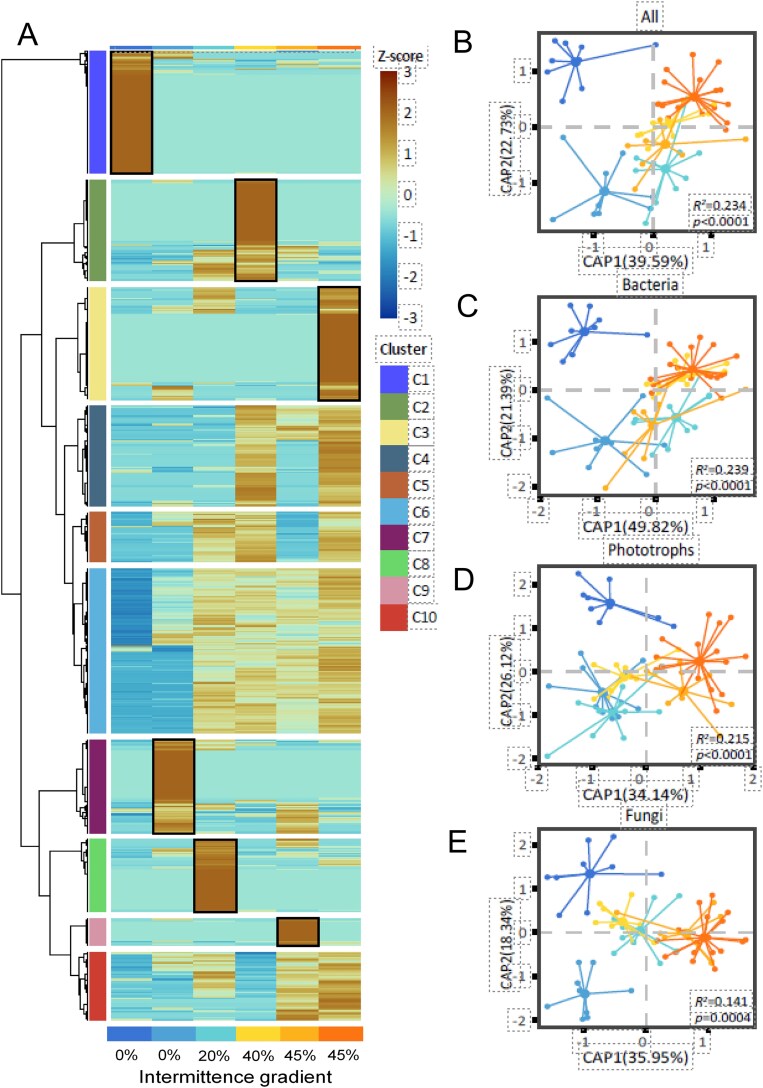
A. The hierarchical clustering heatmap shows the relative abundance (log10-transformed) of each ASV present in the periphyton microbial community across the intermittence gradient (%); black outlines indicate *specific* and *non-specific* clusters (see Table 2); B. Constrained analysis of principal coordinates (CAP) showing the β-diversity calculated as the bray–Curtis dissimilarity average dissimilarity between sites to the centroid of the intermittence gradient (%) for the entire periphyton microbial community, C. The bacterial community, D. The phototrophic community, and E. The fungal community. Variance explained (R^2^) by the intermittence gradient from PERMANOVA is indicated along with the p-value.

To explain how ASVs drive the α-diversity response across the intermittence gradient, we identified six specific clusters through a heatmap that represented unique ASVs for each intermittence gradient, and four non-specific clusters with ASVs occurring throughout the gradient (Fig. 3A). Within specific clusters, Clusters One and Seven exhibited Z-scores greater than 2, suggesting that ASVs in these clusters were substantially more abundant than the average across the intermittence gradient, indicating dominance under 0% intermittence (February and April). Conversely, ASVs with Z-scores under 2 were markedly less abundant, suggesting a reduction in their abundance throughout the intermittence gradient, particularly at 0% intermittence (February and April). In specific clusters, Cluster One at 0% intermittence (February) and Cluster Three at 45% intermittence (December) exhibited the highest number of ASVs, with the main difference being that Cluster One had a higher proportion of bacterial ASVs compared to Cluster Three (Table 2). An even proportion of ASVs between bacteria and phototrophs occurred in Cluster Two at 40% intermittence (August). Cluster Nine had the lowest number of specific ASVs at 45% intermittence (October), and simultaneously, this cluster showed the highest number of fungi ASVs than the rest of the gradient (Table 2). In non-specific clusters, we found the highest number of ASVs in cluster Six (769) with a peculiar separation between ASVs at 0% intermittence (February and April) and the rest of the gradient (Table 2, Fig. 3A).

**Table 2:** Heatmap clusters results of the number of ASVs for the periphyton microbial community and the proportion of ASVs for each group.

**Cluster**	**Type of cluster**	**Intermittence gradient**	**# of ASVs**	**Bacteria (%)**	**Phototrophs (%)**	**Fungi (%)**
C1	Specific	0% (February)	571	91.07	2.98	5.95
C7	Specific	0% (April)	436	63.53	28.44	8.03
C8	Specific	20% (June)	339	52.21	37.17	10.62
C2	Specific	40% (August)	471	43.32	44.37	12.31
C9	Specific	45% (October)	126	55.56	29.37	15.07
C3	Specific	45% (December)	524	60.3	31.11	8.59
C4	Non-specific	all gradient	468	63.25	29.91	6.84
C5	Non-specific	all gradient	231	74.89	19.91	5.2
C6	Non-specific	all gradient	769	74.25	23.41	2.34
C10	Non-specific	all gradient	318	42.77	40.88	16.35

The β-diversity (average dissimilarity) of periphyton microbial communities in the Cube Drying River Network decreased with the increase of drying in the intermittence gradient (*R*^2^ = 23.47%, *p < 0.0001*). The effect of intermittence was highly significant for the β-diversity of the entire microbial community and for each group individually (Fig. 3B-E). However, models showed that >75% of the variation in the data is explained by other ecological factors. We observed that β-diversity, expressed as the average dissimilarity of samples to the centroid within each intermittence gradient, decreased with increasing intermittence. This pattern indicates that periphyton microbial communities became progressively more similar to one another within the same intermittence condition, reflecting community homogenization under higher drying intensity (Figs. 3C, D, and  E). The most remarkable effect of drying was observed in the dissimilarity of the bacterial community, which decreased at 40% and 45% intermittence (December). In contrast, the community dissimilarity within sites at 45% intermittence (October) was highly variable and distinct from the rest (Fig. 3B). The phototrophic community dissimilarity at 0% intermittence (April) and 20% intermittence (June) decreased compared to higher intermittence conditions (Fig. 3C). The fungal community dissimilarity was higher at 0% intermittence (February and April) and decreased with increasing drying (Fig. 3E). All microbial communities at 45% intermittence (October) revealed a greater dissimilarity (dissimilarity to the centroid) than at 45% intermittence (December), indicating that drier conditions induced more variability between sites at the same intermittence gradient (%) in the Cube DRN.

### Hydrological variables and water chemistry effect on periphyton microbial communities

**Figure 4:**
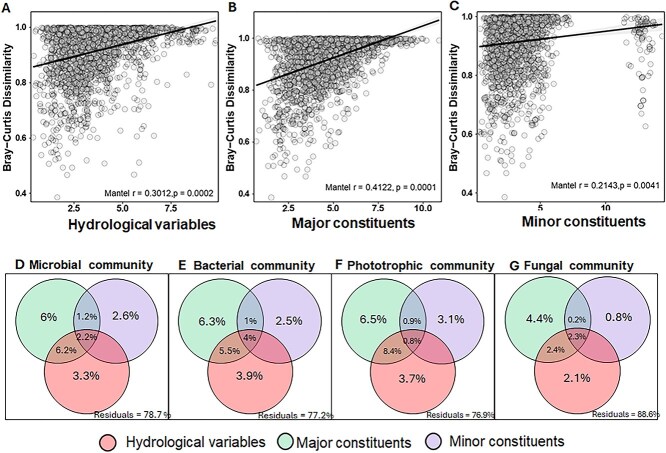
A. Mantel test scatter plots of the periphyton microbial community dissimilarity dissimilarity matrix and environmental variables matrix, B. *Major* dissolved constituents matrix, and C. *Minor* dissolved constituents matrix, simple linear regression *r* indicates the strength of prediction and *P value* indicate the significance after permutations; and Venn diagrams of variance partitioning showing the contribution of each set of variables to explain the D. Microbial community dissimilarity, E. Bacterial community, F. Phototrophic community, G. And fungal community.

To test whether the dissimilarity in the composition (β-diversity) of the periphyton microbial community was correlated to corresponding hydrological variables and water chemistry constituents across the intermittence gradient, we performed a Mantel test between paired dissimilarity matrices (Figs. 4A–C). Although the relationships between the dissimilarity matrices of community composition (β-diversity) and the hydrological variables (*r* = 0.3012*, p = 0.0002*), and major constituents (*r* = 0.4112, *p = 0.0001*) were barely strong, they were statistically significant, supporting the evidence that the two matrices were related in a non-random way (Fig. 4A and B). Minor constituents had also a weak but significant prediction effect on community dissimilarity (*r* = 0.2143*, p = 0.0041*) (Fig. 4C). Since hydrological variables, as well as major and minor constituents in water, acted as surrogate drivers shaping community composition, we performed a variance partitioning analysis to disentangle their relative contributions. We found that the major constituents alone (i.e., Ca^2+^, Mg^2+^, NO^3−^, and TOC) (Table S2) explained ~6% of the variance in the periphyton microbial community and across all groups (Fig. 4D). Hydrological variables (i.e., Temperature, Depth, Discharge) (Table S1) and minor constituents (i.e., Al^3+^, Fe^2+^, Cu^2+^) (Table S3) contributed to explaining ~3% and 2% of the variation (Fig. 4E and F). The fungal community had the lowest explained variance, with major constituents accounting for 4.4%, environmental variables for 2.1%, and minor constituents for only 0.8% (Fig. 4F). After assessing the residual variance, we found that microbial communities remained highly unexplained (76.9%–88.6%) (Fig. 4). Together, these results indicate that while hydrological variables and water chemistry are significantly associated with periphyton community dissimilarity, their explanatory power is limited relative to the overall variability observed across the intermittence gradient.

### Ecological co-occurrence interactions in the intermittence gradient

**Figure 5:**
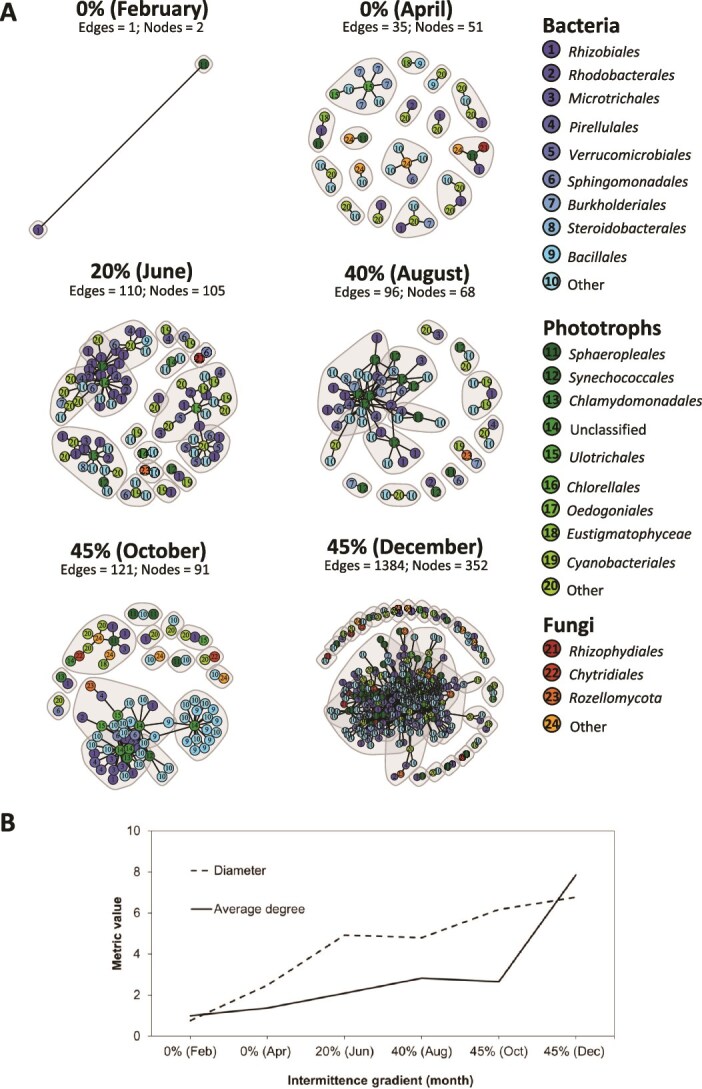
A. Ecological co-occurrence networks of periphyton microbial communities in the cube Drying River network across the intermittence gradient (%). Co-occurrence networks show 1% of positive ecological interactions between bacteria, phototrophs, and fungi. Node numbers represent order-specific taxonomy: Bacteria, phototrophs, and fungi. Edges indicate whether the interactions are strong (nearby nodes) or weak (distant nodes). Orders in the legend are ordered such that they represent higher or lower node abundance across the intermittence gradient (i.e., *Pirellulales* has higher node abundance than *Verrucomicrobiales*; *Sphaeropleales* is the phototrophic order with the most nodes, followed by *Cyanobacteriales*); B. The trend graph shows the increase in diameter and average degree with the intermittence gradient.

We created six co-occurrence networks assessing the dynamics of interactions under each intermittence condition (Fig. 5A). Our results showed an increase in all network metrics, indicating a higher number of groups, links, and nodes with the increase in intermittence (Table 3, Fig. 5B). We found that the number of communities (tightly connected groups) was higher, and modularity was lowest at 45% intermittence (December) compared to the rest of the co-occurrence networks under different intermittence conditions (Fig. 5B, Table 3). This means that 28 microbial groups co-occurred, sharing similar habitat requirements with a strikingly high number of links (1384). Interestingly, the subsequent high number of communities (17) occurred at 20% intermittence (June), with a clear dominance of bacteria, including *Rhizobiales*, *Rhodobacterales*, and *Microtrichales*, as well as mostly *Cyanobacteriales* from the phototroph community (Fig. 5A). Conditions at 0% intermittence (February) allowed a poor co-occurrence network with only two nodes: *Rhizobiales* and *Sphaeropleales*. However, at 0% intermittence (April), the high modularity and the presence of 16 communities suggested that as soon as suitable conditions resumed, with reduced shear stress and stable flow conditions, the community increased in complexity (Table 3).

**Table 3:** Co-occurrence networks metrics of periphyton microbial communities in the intermittence gradient.

**Intermittence gradient**	**# of communities**	**Modularity**	**Mean distance**	**# of links**	**# of nodes**	**Bacteria (%)**	**Phototrophs (%)**	**Fungi (%)**
0% (February)	2	0.0000	0.7615	1	2	50	50	0
0% (April)	16	0.9034	1.2850	35	51	45.71	42.86	11.43
20% (June)	17	0.7447	2.3817	110	105	50	49.09	0.91
40% (August)	14	0.4942	2.2034	96	68	49.48	49.48	1.04
45% (October)	15	0.5528	2.7442	121	91	46.28	48.76	4.96
45% (December)	28	0.4105	2.4032	1384	352	48.81	47.76	3.43

At 40% intermittence (August), the co-occurrence network exhibited the lowest modularity, alongside the 0% intermittence (February), with fewer links and nodes, and only 14 communities. Overall, co-occurrence networks at 0% intermittence (February and April) had the lowest diameters, indicating the highest degree of total connectedness between the two communities. In contrast, at 45% intermittence (October and December), the larger diameter suggests less direct interaction between fragmented groups (Fig. 5B).

## Discussion

In this study, we found that microbial community composition in the periphyton varied in response to intermittence: α-diversity increased with higher intensity, and at the peak of the intermittence (45% - December), the relative abundance of bacteria, phototrophs, and fungi groups changed with the intermittence gradient, with a marked effect on phototrophs. The observed decrease in β-diversity with increasing intermittence reflects reduced within-level community dispersion rather than differences among intermittence levels, suggesting that prolonged hydrological fragmentation promotes convergence of periphyton microbial communities. Our results allow us to entirely reject the stated hypothesis **H1**, as α-diversity did not decrease with drying in tropical DRNs. The relative abundance was characterized by more groups (>5%) rather than fewer at the drier end of the intermittence gradient. We also found that an increase in the intermittence gradient allowed for a higher abundance of specific ASVs and related to the increase in α-diversity. However, more non-specific ASVs are associated with a more homogeneous community, resulting in a decrease in β-diversity. Although periphyton represents a surface-attached microbial community that forms a distinct microenvironment from the planktonic compartment, it remains tightly coupled to the surrounding water column through continuous exposure to dissolved constituents. Thus, hydrological variables and water chemistry were expected to act as modulators rather than primary determinants of periphyton microbial composition under variable intermittence conditions (**H2**). Our results partially support this hypothesis, as major dissolved constituents explained a small but significant fraction (~6%) of community variation, while most variance remained unexplained. This highlights the importance of the co-occurrence of taxa within the periphyton matrix, particularly in relation to the potential uptake of distinct constituents. Additionally, environmental variables could be paired with biotic interactions that exert distinct pressures on different resources in the periphyton (i.e., grazing). Indeed, we found that changes in community composition coincided with more complex interactions within the periphyton as intermittence increased. This result allows us to reject hypothesis **H3**, as we expected the interactions to decrease under highly hydric stress conditions.

### Drying in a tropical drying river network triggers changes in the microbial community composition

We found that tropical DRNs undergo hydrological fragmentation and surface disconnection, rather than experiencing fully exposed dry beds [1, 3]. In this sense, our results contrast with previous research conducted in other latitudes, which shows that extended drying episodes impose harsh conditions on periphyton (i.e., algal, prokaryotic, and fungal communities) [8, 11, 26] and other aquatic organisms [5–8, 10, 24]. However, our intermittence gradient may align with previous results [27], which describe an incomplete drying pattern in which lotic, lentic, and terrestrial habitats co-occur sequentially along a longitudinal gradient [2, 3, 5]. Our intermittence gradient at the river network scale was characterized primarily by extended periods of stable, low-flow conditions, followed by a predominance of isolated pools rather than dried sites [22]. In this sense, the intermittence gradient captured more accurately the drying process, whereby reduced-turbulence habitats likely facilitated the settlement and attachment of new communities at 20% intermittence (June) and potentially activated dormant organisms [9, 11, 14, 28, 45]. These conditions likely allowed periphyton to encroach on emerging habitats, reflecting a synergistic cue between reduced shear stress and the lag in particle settlement that boosted the growth of all groups (Fig. 2) [20, 25, 27, 46, 47]. Additionally, not only the presence of shallow waters in isolated pools and barely deep-water columns during residing flows, at 20% intermittence (June) onwards, favored bacteria and phototrophs (Fig. 2), but we observed that upon this intermittence gradient, the Cube DRN experienced the highest incoming radiation measured at the headwaters of the DRN (Fig. FS1). Therefore, we can explain the blooming of phototrophs like Chlorophyta (i.e., *Sphaeropleales*), Gyrista (i.e., *Naviculales*), and Cyanophyta (i.e., *Cyanobacteriales*) [46, 48] that are known to stabilize substrates, serving as habitats for other groups and many superior organisms (i.e., *Chironomidae*) (Fig. 2D) [24, 29, 49, 50].

The striking difference that we found between the community composition at the appearance of drying sites, 20% intermittence (June), with conditions during flashy floods (Fig. 1C) [51] in February and April, is not particular to Drying River Networks, but accurately explained the highly seasonal hydrological regime in a Tropical system (Fig. FS1) [2, 3, 22]. During high-flow conditions, only strictly anaerobic bacilli with adhesive holdfasts, such as *Caulobacter* and *Clostridiales*, and orders that sporulate under adverse conditions, like *Bacillales* and *Paenibacillales*, resisted scouring, suggesting that under high flows, microbial periphyton activity is orchestrated by bacteria (Fig. 2B) [46, 52–54]. However, because we predominantly observed an intermittence gradient, conditions between February and April (0% intermittence) differed, providing strong evidence that preceding flow variability is important in explaining the intermittence intensity.

### Drivers of periphyton microbial community composition

Across the Cube DRN, hydrological intermittence emerged as the primary structuring force of periphyton microbial communities, integrating physical fragmentation, reduced shear stress, and altered habitat connectivity. Within this intermittence-driven framework, water chemistry and hydrological variables functioned as secondary or permissive filters rather than primary determinants of community assembly. Major dissolved constituents (e.g., Ca^2+^, Mg^2+^, NO₃^−^, TOC) explained a small but significant fraction (~6%) of community variation, suggesting that chemical conditions modulate community composition once physical intermittence constraints are established [55]. Upon the start of drying, at 20% intermittence (June), hydrological variables and water chemistry stabilized, providing conditions for a diverse pool of microbial groups [56]. The response to drying conditions suggests that specific clusters comprising 58% of ASVs were driven mainly by the intermittence gradient.

The relatively high proportion of unexplained variation further reflects the intrinsic complexity of periphyton biofilms, where micro-scale heterogeneity, internal nutrient recycling, and biotic interactions can decouple microbial assemblages from bulk water conditions [14, 17, 47, 53, 55, 56]. In this context, it is also important to acknowledge that while all samples were initially processed using the same commercial DNA extraction kit, a subset of periphyton samples required phenol–chloroform extraction to obtain sufficient DNA from the biofilm matrix. Although differences in extraction chemistry can influence fine-scale taxonomic recovery, these samples did not exhibit systematic differences in community composition, diversity metrics, or ordination patterns. This supports the robustness of the observed ecological patterns, particularly the hierarchical dominance of intermittence over secondary environmental drivers, while suggesting caution when interpreting subtle taxonomic shifts.

### Increments in periphyton microbial interactions diminish network resilience under an intermittence gradient

While community composition became more diverse and structurally reorganized with increasing intermittence, reflected by higher α-diversity and shifts in relative abundance among microbial groups, these patterns indicate compositional redistribution rather than increases in absolute abundance. Our analysis revealed a high number of links with an increase in intermittence and, simultaneously, a decrease in modularity (Table 4), indicating that each co-occurrence network was highly connected within the network but possibly ecologically isolated from other communities due to fragmentation resulting from drying [53, 54]. This corroborates our previous finding on decreasing community dissimilarity with drying, suggesting that communities have similar ecological roles and feeding habits and are influenced similarly by common environmental factors. We found an even number of nodes representing close interactions between bacteria and phototrophs, indicating low compartmentalization in the networks [42, 53, 54]. These results align with the plasticity observed in periphyton, where bacteria and fungi generally exhibit greater resistance to hydric stress than phototrophs [51, 53]. The persistence of these stress-tolerant groups under intermittent conditions likely maintains biofilm structure and internal nutrient cycling, creating a stable metabolic framework that facilitates tighter coupling with phototrophs when light and water availability permit [57, 58]. Consequently, the increased strength of bacteria–phototroph interactions observed under higher intermittence likely reflects indirect facilitation mediated by carbon exchange, nutrient retention, and substrate stabilization rather than a direct effect of stress resistance alone [57, 59]. Fungal nodes were scarce in co-occurrence networks across the intermittence gradient, suggesting a possible secondary input of fungal organisms in shaping the microbial composition and diversity. However, their ecological role should not be underestimated, particularly for parasitic chytrids (Chytridiomycota) [59–61]. *Rhizophydiales* and *Chytridiales* (Fig. 5) were the two largest orders from the Chytridiomycota phylum, both of which have been documented as being algae parasites [62, 63], Their importance tended to increase as drying conditions intensified, and greater α-diversity was observed; this fungal diversification might be influenced by the parallel increase in α-diversity detected in phototrophic organisms under the same intermittent conditions.

Characterizing microbial communities living in stream periphyton through ASVs revealed a previously undocumented taxonomic diversity and complexity of community structure over changing environmental conditions, providing important insights into the strategic dynamics of organisms facing drying conditions and streamflow variations. Our spatiotemporal research on the Cube DRN demonstrates the modulating effect of intermittence on enhancing stream periphyton microbial communities in a global biodiversity hotspot. Additionally, considering the hypotheses and observed patterns, the intermittent gradient in the Cube DRN differed from those in other latitudes, as microbial α-diversity increases, and β-diversity decreases with drying. This suggests that despite the potential of periphyton communities to thrive in dry conditions in the tropics, they face disturbances with the same set of skills. The implications of these results underscore the importance of further research on the functional processes associated with the observed diversities, which may be at risk given the expected increase in non-perennial segments worldwide [4, 5]. As the resilience of microbial communities in the periphyton is already vulnerable under natural intermittence, an increase in non-perennial segments poses a new set of challenges for tropical river networks experiencing drying.

## Supplementary Material

SupplementaryMaterial_Microbial_Intermittence_ycag084

## Data Availability

All sequences are available under Bioproject ID: PRJNA1436123 http://www.ncbi.nlm.nih.gov/bioproject/1436123 and Bioproject ID: PRJNA1436150 http://www.ncbi.nlm.nih.gov/bioproject/1436150. All codes are available in 10.5281/zenodo.18975553.
