# Supplementary material for: Spatiotemporal intermittence effect on periphyton microbial communities in a tropical drying river network
Source: ISME Commun. 2026 Apr 16;6(1):ycag084. doi: 10.1093/ismeco/ycag084 (PMC13137328; doi:10.1093/ismeco/ycag084)
Supplement: SupplementaryMaterial_Microbial_Intermittence_ycag084 [file supplementarymaterial_microbial_intermittence_ycag084.docx]

**Title:** **Spatiotemporal intermittence effect on periphyton microbial communities in a tropical drying river network**

Milena D. Campaña^1^, Daniela Rosero-López^1,2*^, María de Lourdes Torres^2,3^, Daniel

Escobar-Camacho^1,2^, J.L. Weissman^4,5^, Juan José Guadalupe^3^, Darío X. Ramírez-Villacís^3^, Jordan Karubian^6,7^, Andrea C. Encalada^1,2^

**Affiliations**

^1^ Laboratorio de Ecología Acuática, Universidad San Francisco de Quito, Diego de Robles S/N, Quito, Ecuador

^2^ Global Research and Solutions Center, Universidad San Francisco de Quito, Diego de Robles S/N, Quito, Ecuador

^3^ Laboratorio de Biotecnología Vegetal, Universidad San Francisco de Quito, Diego de Robles S/N, Quito, Ecuador.

^4^ Department of Ecology and Evolution, Stony Brook University, Stony Brook, NY, USA

^5^ Institute for Advanced Computational Science, Stony Brook University, Stony Brook, NY, USA

^6^ Department of Ecology and Evolutionary Biology, Tulane University, New Orleans LA, 70118, USA

^7^ Fundación para la Conservación de Los Andes Tropicales, Quinindé, Esmeraldas Province, 080451, Ecuador.

**Supplementary Methods**

We incubated the samples with solution CD1 for 10 minutes at 70°C. For the bead-beating step, we placed samples vertically in a vortex at max speed for 40 minutes. After adding solution CD2, we resumed the manufacturer’s protocol. Finally, we used 45 μL of solution C6 for the final DNA elution. We measured DNA concentration and quality using a NanoDrop-2000 spectrophotometer (Thermo Scientific®). A phenol-chloroform DNA extraction protocol was conducted for samples that showed low DNA concentration and quality after the first extraction, adapted from Busi et al. (2020). After recovering 500 ml of pellet from every 7.5 ml of sample, we added 800 μl of lysis buffer (0.1M Tris-HCl, 0.05 M EDTA, 1.5% SDS; pH 7.52) and 2.5 μl of RNase A (20 mg/ml). After vortexing for 15 seconds, the samples were incubated at 37°C for 1 hour and homogenized every 15 minutes. Subsequently, we added 6.66 μl of Proteinase K (15 mg/ml), and then we incubated the samples at 70°C for 10 minutes. After a brief spin, we added 750 μL of Phenol:Chloroform:Isoamyl Alcohol (25:24:1) to the tubes. The contents were thoroughly mixed and centrifuged at 7.500 g at 4°C for 15 minutes. Then, we transferred the aqueous phase to a new tube and added the same volume of chloroform and isoamyl alcohol (24:1). We mixed the tubes vigorously, rested for 10 minutes, and then centrifuged at 7,500 g at 4°C for 15 minutes. We transferred the aqueous phase to a new tube, adding the same volume of isopropanol. The tubes were mixed by inversion and incubated at -20°C overnight. The next day, we centrifuged the samples at 12.000 g at 4°C for 35 minutes. We discarded the supernatant and washed the pellet once with 500 μL of 70% ethanol. Ethanol was carefully removed, and the pellet was left to dry completely. Subsequently, we dried the pellet and resuspended it using the CD6 solution provided in the DNeasy PowerSoil Pro DNA Isolation Kit (Qiagen®, USA). We purified the DNA using AmpureXP magnetic beads (Beckman Coulter) at a 1.8X concentration.

**Supplementary Figures**

**Figure FS1.** Representative water level and solar radiation in the Cube Drying River Network depicting the wet and dry season (a) and the peak of incoming light (b).


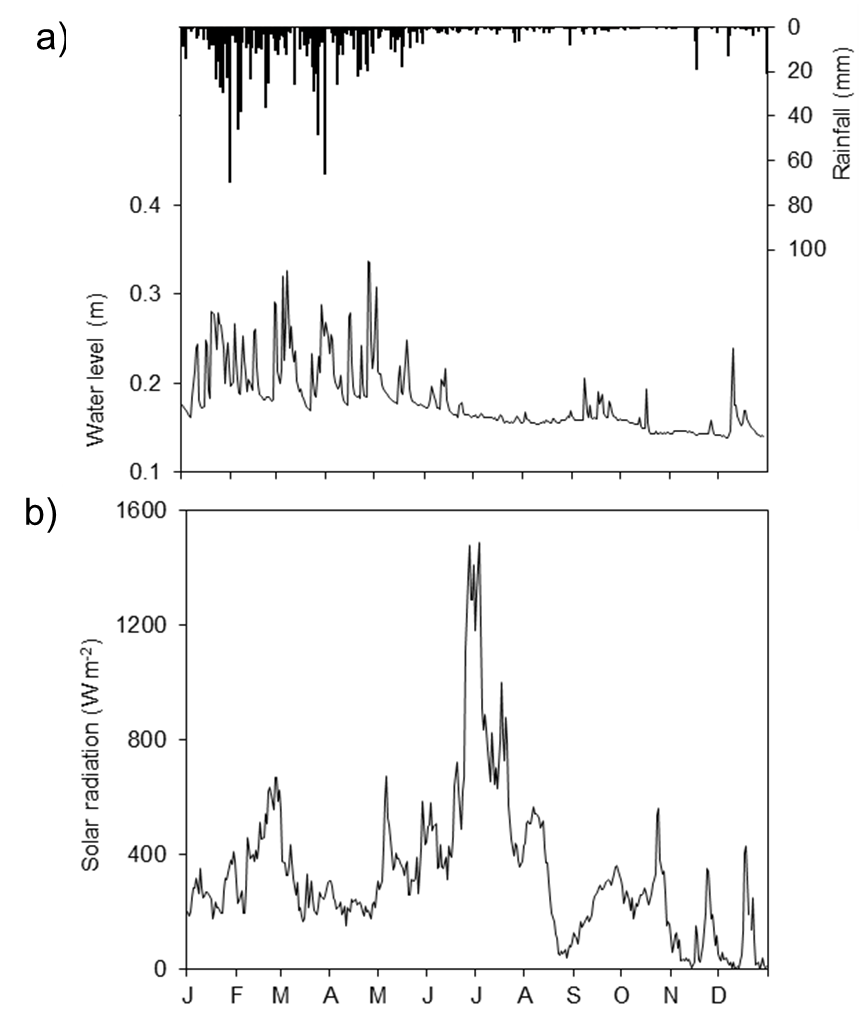


**Figure FS2.** Rarefaction curves by sampling campaign: M1 – February; M2 – April; M3 – June; M4 – August, M5 – October; M6 – December.

**
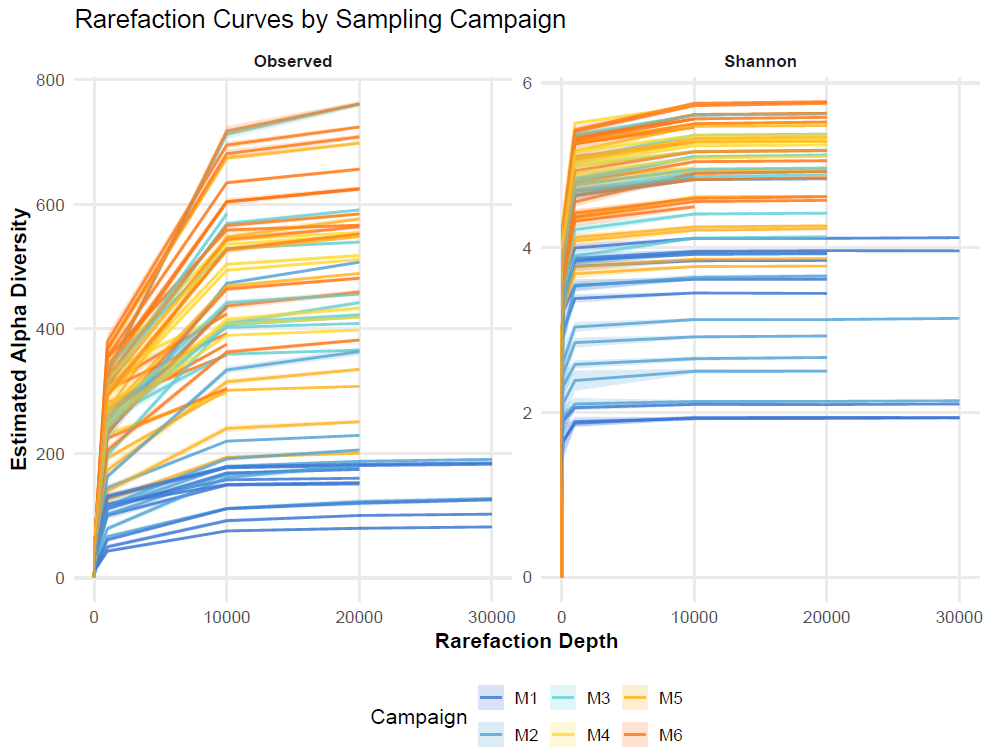
**

**Supplementary Tables**

**Table S1.** Hydrological variables measured in the Cube Drying River Network (n=20) in six sampling campaigns (mean±SD) responding to an intermittence gradient.

**Table S2.** Major constituents present in water samples from sites (n =20) during six sampling campaigns responding to the intermittence gradient.

**Table S3.** Minor constituents present in water samples from sites (n =20) during six sampling campaigns responding to the intermittence gradient.

**Table S4.** Unpaired two-sided t-tests of α-diversity between intermittence phases


**Table S5.** Filtration of samples to keep only high-abundance ASVs (> 0.1% relative abundance).
